# Deep learning and generative methods in cheminformatics and chemical biology: navigating small molecule space intelligently

**DOI:** 10.1042/BCJ20200781

**Published:** 2020-12-08

**Authors:** Douglas B. Kell, Soumitra Samanta, Neil Swainston

**Affiliations:** 1 Department of Biochemistry and Systems Biology, Institute of Systems, Molecular and Integrative Biology, Faculty of Health and Life Sciences, University of Liverpool, Crown St, Liverpool L69 7ZB, U.K.; 2 Novo Nordisk Foundation Centre for Biosustainability, Technical University of Denmark, Building 220, Kemitorvet, 2800 Kgs. Lyngby, Denmark

**Keywords:** artificial intelligence, cheminformatics, deep learning

## Abstract

The number of ‘small’ molecules that may be of interest to chemical biologists — chemical space — is enormous, but the fraction that have ever been made is tiny. Most strategies are discriminative, i.e. have involved ‘forward’ problems (have molecule, establish properties). However, we normally wish to solve the much harder generative or inverse problem (describe desired properties, find molecule). ‘Deep’ (machine) learning based on large-scale neural networks underpins technologies such as computer vision, natural language processing, driverless cars, and world-leading performance in games such as Go; it can also be applied to the solution of inverse problems in chemical biology. In particular, recent developments in deep learning admit the *in silico* generation of candidate molecular structures and the prediction of their properties, thereby allowing one to navigate (bio)chemical space intelligently. These methods are revolutionary but require an understanding of both (bio)chemistry and computer science to be exploited to best advantage. We give a high-level (non-mathematical) background to the deep learning revolution, and set out the crucial issue for chemical biology and informatics as a two-way mapping from the discrete nature of individual molecules to the continuous but high-dimensional latent representation that may best reflect chemical space. A variety of architectures can do this; we focus on a particular type known as variational autoencoders. We then provide some examples of recent successes of these kinds of approach, and a look towards the future.

## Introduction

**Figure 1.**
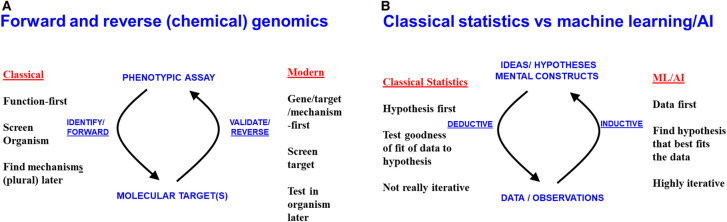
Two ways of relating paired attributes lead to separate strategies depending on the starting point. (**A**) Forward and reverse chemical genomics. (**B**) Classical statistics vs machine learning. Note that Bayesian statistics is much closer in spirit to machine learning.

Much of chemical biology is involved with the study of the interactions between small molecules and biomacromolecules, along with any physiological consequences, usually with the aim of finding molecules that are in some senses ‘better’. At a high level, this admits two strategies [1] (Figure 1A). The classical version of chemical genomics was data-driven or ‘function first’; a small molecule was applied to the system of interest (e.g. a rodent inoculated with *Mycobacterium tuberculosis*) and it either worked (here to kill the bacteria) or it did not. No mechanistic understanding was required (though could later be sought). A major advantage was, after all, that the drug worked. Beyond the thought of trying a variety of molecules, no specific hypothesis was required. In a more modern version, a target (or, much more occasionally a set of targets) is sought, on the basis of a hypothesis, usually about the desirability of inhibiting said target, and typically on a purified protein *in vitro*. Following a terminology from genetics, the former is referred to as ‘forward’ chemical genomics, the latter as ‘reverse’ (Figure 1A). The nominal advantage of the reverse approach is that in theory one immediately has a mechanism. However, even this is illusory, as effective drugs normally have multiple targets [2], and the ability to bind to a target *in vitro* conveys little or nothing about its mechanisms, efficacy or toxicity *in vivo* [3], nor even if it can even reach the supposed target(s) (membrane transporters are normally involved [3–7]). Thus, the search for a molecule with desirable properties (however defined) typically involves a cyclic interplay of the type implied in Figure 1A. As with protein optimisation [8], it is arguably best seen as a navigation through a large search space of possible solutions [9].

Recently, there has been much excitement about the use of methods referred to as ‘Machine Learning’, ‘Artificial Intelligence’, or simply ML or AI. These too can serve to relate the world of ideas to the world of data. Perhaps surprisingly, their relationship to classical (Neyman-Pearson or frequentist) statistics [10] (Figure 1B), is similar to that between forward and reverse chemical genomics (Figure 1A).

### Chemical space

**Figure 2.**
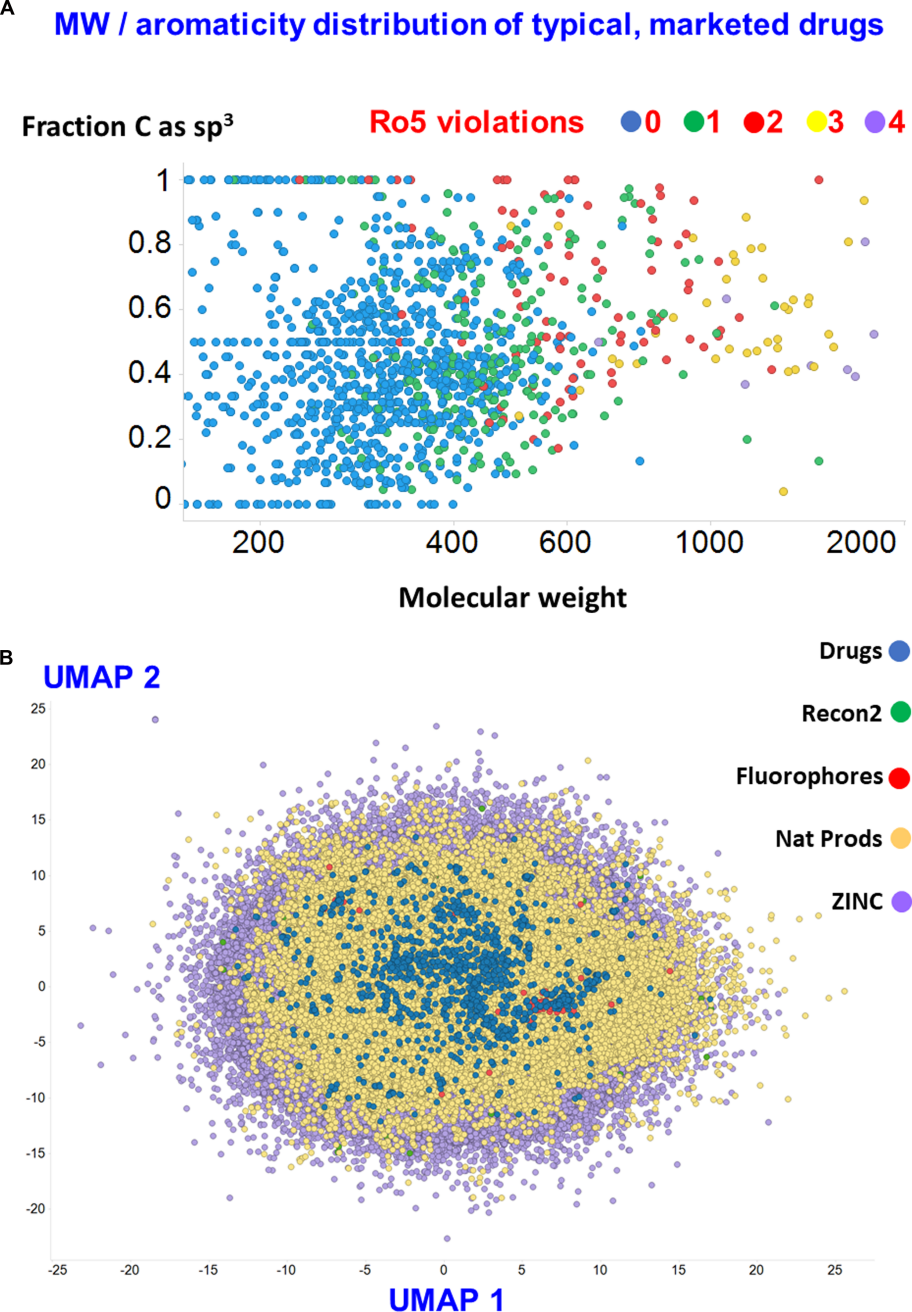
The areas of chemical space presently occupied by some 1387 marketed drugs. (**A**) Fraction of sp^3^ hybridisation (a measure of aromaticity) plotted vs molecular weight. The number of violations of the ‘Rule of 5’ [23] is also shown. (**B**) UMAP [24] representation of the chemical space of ∼6 M ‘druglike’ molecules from ZINC. This is largely seen to contain the ∼150 000 natural products, ∼150 fluorophores, ∼1100 endogenous human metabolites (Recon2) and 1387 marketed drugs studied previously [25]. Molecules were extracted by the present authors [26] to a latent space of 100 dimensions using methods described in [27] and their vector values in the latent space used as the input to the UMAP algorithm.

The essential problem is that the number of small molecules of potential interest (‘chemical space’) is vast [11–13]. A widely quoted figure, based on simple calculations, albeit dependent on the number of C-atoms considered, is 10^60^ [14,15]. In contrast, the numbers of synthesised and purchasable molecules as recorded at the ZINC database [16] (http://zinc15.docking.org/) are just over 10^9^ and 6.10^6^, respectively (even most of the simple heterocyclic scaffolds have never been made [17,18]). Restricting the number of heavy atoms to just 17, including halogens as well as C, N, O and S, gives more than 10^11^ molecules [19,20]. This corresponds to an average molecular weight (MW) ∼250 [19], while MWs of 500 and 1000 imply ‘Universes' (for even these restricted chemical spaces) of ca 10^36^ and ∼10^72^, respectively [15]. An earlier list of 1387 marketed drugs [21] includes over 200 of them (some 15%) with MW exceeding 500 (Figure 2A), while a 2D mapping of ∼6 million ZINC compounds, 150 000natural products, ∼150 fluorophores, ∼1100 endogenous human metabolites (Recon2), and the same marketed drugs (based on [22]) is given in Figure 2B.

Recent advances in computational learning have changed the game of how we can understand and navigate this very large chemical spaces, from a focus on **discriminative**^1^
^1^**Terms in boldface** are defined further in a glossary at the end methods, that are largely descriptive, to a suite of **generative** methods in which we develop and deploy the ability to create novel matter computationally and in principled ways. The purpose of this review is to describe these changes.

## A brief history of virtual screening and the multilayer perceptron

Because of these very large numbers, that far exceed the capacity of even high-throughput screening assays, virtual screening (VS) has come to the fore. VS [28–31] refers to the use of computational techniques to explore a large compound library *in silico*, thereby to select a small subset of potentially interesting (here bioactive) molecules that may then be tested experimentally. It is useful to discriminate ‘**unsupervised**’ from ‘**supervised**’ learning methods, where in the former we know only the inputs, e.g. molecular structures, without knowing the outputs, e.g. activities of interest. Complementarily, semi-supervised or ‘self-supervised’ [32,33] methods leverage knowledge of the output values (or class labels) where they are known for a subset, while reinforcement leaning methods involve the use of machine learning methods to make decisions based on various kinds of reward. Although VS covers many areas, we shall focus here on Quantitative structure-activity relationships (QSAR) (e.g. [34–36]). QSAR describes a series of techniques for supervised learning, in which we use paired inputs (X) and outputs (Y) (here they are suitably encoded molecular structures and activities, respectively) to produce a model that given the former outputs the latter.

**Figure 3.**
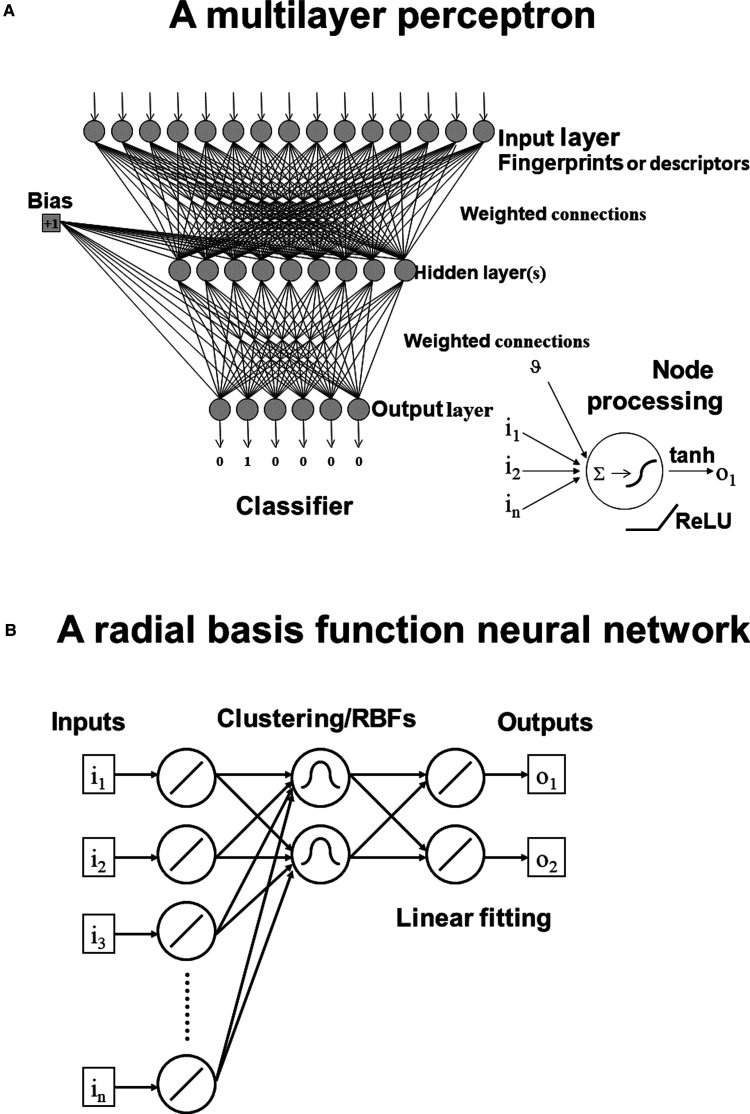
Early, ‘shallow’ neural networks. (**A**) A fully interconnected feedforward network: the multilayer perceptron. In the lower right is shown the action of a processing unit as summing its inputs plus the bias ν then passing them through a transfer function to the next node. Usually this transfer function was a saturable, differentiable function such as tanh, but more recently ‘rectified linear' nonlinearities have become popular. The example shown is a classifier, though outputs can be non-integer numbers and the ANN serve as a nonlinear regressor. (**B**) A radial basis function neural network. Here the first step is an unsupervised clustering of the input data (typically using K-means clustering) that sets the centres and widths of the radial basis functions according to the local density of points in the input space. A simple linear fitting then determines the outputs.

One such means of effecting this mapping is the multilayer perceptron (MLP), in the form of a fully interconnected feedforward artificial neural network [37] (Figure 3A). The MLP consists of nodes (circles) and weights (the lines joining them). Typically in chemical biology and QSAR analyses the inputs (X, just 15 are shown in Figure 3A) would be the values of molecular descriptors or the digits of a binary fingerprint encoding of molecular structure [38]. Outputs (Y) can either be classes or values to be found via a composite nonlinear/mapping function. In the example shown we have six classes. The weights are initialised to small values (typically this was done randomly from a normal distribution; nowadays it is done in a more principled way [39,40]), and a bias term introduced as shown (Figure 3A).

Training such an MLP consists of applying the inputs (usually normalised, commonly in the range 0.1 to 0.9 or 0 to 1 for each) and multiplying them by the relevant weight. A node (shown in the lower right-hand portion of Figure 3A) is a very simple processing unit: it sums the dot product of weights and inputs, then passes it through a transfer function. Classically (in the 1980s/1990s) the transfer function was differentiable, usually something like a hyperbolic tangent function (*tanh*), that scaled inputs to the range 0 to 1 and passed them to the following layer. In Figure 3A only one ‘hidden’ layer is shown before the output layer. In classification problems it is common to use a *Softmax* function at the output layer to ensure that the sum of the outputs is 1, thus producing a result vector that represents the probability distribution of the potential outcomes. Training the networks (finding the ‘best’ values for the weights) typically involves an algorithm (‘backprop’) that propagates a partial derivative of the normalised error between the desired and discovered outputs back through the MLP, the weights being adjusted up and down until the outputs have been learned (i.e. can be reproduced when the input is passed forward through the network). Training can be done in batches using subsets of the paired (X-input, Y-output) training data, and each full pass backwards and forwards through the training set is typically referred to as an epoch. Because such networks (of arbitrary size) can learn any arbitrary mapping (known as ‘**universal approximation**’) they are very prone to **overtraining** (learning the training set but failing on new inputs), and a separate validation set is used to see when this occurs (so as to prevent it). Finally, a completely independent test set is used that has not been used in training at all. This avoids the (correct) criticism by frequentist statisticians that these methods are effectively testing 1000s of hypotheses in a desperate attempt to try and find one that fits [41].

The key concepts of any kind of learning of this type are that (i) such an MLP can provide sensible outputs on molecules it has never seen (this is referred to as ‘generalisation’), and (ii) the ‘knowledge’ that it has ‘learned’ is effectively stored in the matrix of learned weights. In contrast with say a computer memory, this memory is not held in a single location but in the whole network. This idea (which is also adopted in living organisms) is known as ‘associative’ or ‘content-addressable’ memory, and is the main reason for the ‘robustness’ of structures such as MLPs; destroying some of the connections has little adverse effect on them (and may even improve them — see below). Because the MLP model has learned the general mapping of inputs to outputs by storing it in the matrix of weights, any other reasonable molecule can be applied to it and a suitable output will emanate. This permits any kind of virtual screening for which one has a molecular structure that can be encoded as an input and the potential activity can then be output. Note that here we still have to provide all the molecules as inputs.

While the numbers of inputs and outputs are fixed, there are a number of so-called **hyperparameters** of an MLP or similar neural network that can improve the speed and effectiveness of learning and generalisation. For the MLP these include the number and size of the hidden layers, the transfer function, the means of weight initialisation, the learning rate, and the so-called momentum (which adds a fraction of the previous weight update to the current one). In addition, one could usefully remove individual nodes or weights that did not seem to contribute to the model [42,43].

Although such MLPs could indeed be used for virtual screening (and many other purposes), they were very slow to train (**radial basis function networks** [44,45] (as in Figure 3B) were far quicker [46,47]), and it proved impossible to train large nets with even modest (>2) numbers of hidden layers. It is widely considered that this was simply due to the fact that the gradient fed back during the backpropagation step was increasingly small as the number of weights increased (the ‘vanishing gradient’ problem). In other circumstances the gradient could become unstable (the ‘exploding gradient’ problem). Actually, the success of RBF nets gave a strong hint that an unsupervised step prior to (or as part of) the initial weight setting, especially if done layer by layer [48], could be very advantageous.

Another popular neural network architecture of the time was the self-organising feature map popularised by Teuvo Kohonen [49]. It bears some similarities to RBF networks in that it too uses unsupervised methods to cluster the input space prior to the training steps.

At all events, during the 1990s, because of the inability to train large and deep networks, the entire ANN field largely went into stasis save for the activities of a small number of pioneers.

## The rise of deep learning in the 21st century

Obviously, this has all changed, since it is now entirely possible to train ANNs that are broadly similar to MLPs but that can have even hundreds of ‘hidden’ layers (e.g. [50]), totalling over a billion interconnections, which is why they are called ‘deep networks’. These very large networks are the basis for the revolution known as ‘deep learning’ [51–53] that underpins widely recognised advances in image and speech recognition and the ability [54] to play board games at absolutely world-leading levels. At the time of writing (September 2020), probably the largest is GPT-3, containing as many as 170 billion weights [55]. Deep learning is now coming to the fore in drug and materials discovery [56–65]. As set out by Geoffrey Hinton, one of those neural network pioneers, the reasons for the original failure were essentially fourfold: (i) the labelled datasets were thousands of times too small; (ii) typical computers used were millions of times too slow; (iii) the weights were initialised in a naive way; (iv) the wrong types of transfer function (non-linearity) were used.

Thus, one major problem with the *tanh* transfer function (Figure 3A) is that it cannot extrapolate beyond the range on which it has been trained, whereas a rectified linear unit (ReLU) type of transfer function can [66]. This kind of transfer function (Figure 3A), that is zero for all inputs below and including zero and then linear with the sum of the inputs for positive values, is usually far more effective [67], and many others can work well too (e.g. [68,69]). The type of initialisation has also been much improved [39,40], especially using weight initialisations that are based on the size and position of the layer they are in. Finally, the advent of GPU and cloud-based computing has made very powerful computers much more widely available. With this has come the recognition that not only can deep nets ‘store’ more knowledge than can shallow nets, but that they require to be trained on many more input examples. Such large datasets are nowadays commonly available online; when they are not, a useful and effective trick of data augmentation is to add certain kinds of ‘noise’ to those that are [70,71]. This said, a variety of machine learning methods are perfectly capable of learning large datasets for ‘discriminative’ QSAR problems with more or less equal facility [72], and deep learning methods are likely to have only a marginal advantage [73]; a particularly detailed comparison has recently appeared [74].

Although the basic MLP architecture could be modified, it rarely was, and one of the other main features of the deep learning renaissance is the coming to prominence of a series of other architectures. We shall discuss four broad classes: convolutional (CNNs), recurrent (RNNs), long short-term memory (LSTMs), and auto-associative (AA) nets. This will lead us on the variational autoencoder that is our focus here.

## Convolutional neural networks (CNNs, ConvNets)

**Figure 4.**
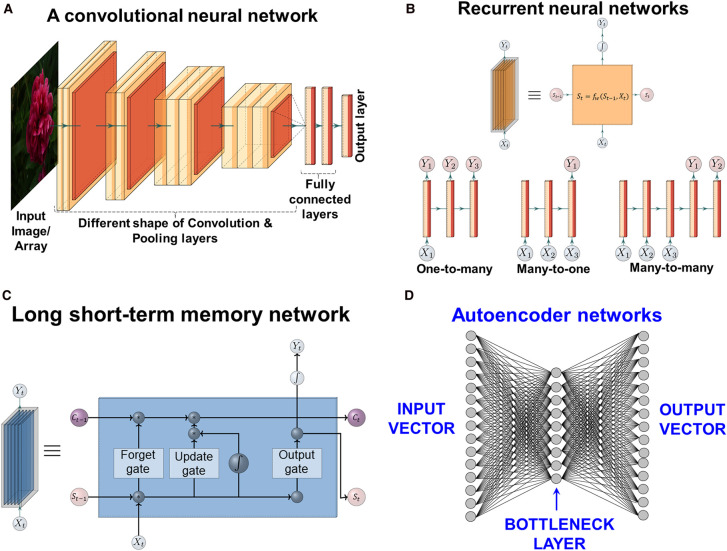
Networks with other architectures. (**A**) Convolution neural networks (CNNs, ConvNets). (**B)** Different types of recurrent neural nets (RNNs) based on their input–output size. The upper row shows a general overview of an RNN unit with different components and the lower row shows the different types of RNNs. (**C**) Long short term nets (based on [83] and [84]), showing the architecture of the LSTM neuron and three gate units. The self-recurrent connection on the left indicates the feedback with a delay of one time step. c_t−1_ and c_t_ are the contents of the cell at time t−1 and t, while s_t−1_ and s_t_ represent the network state. For further details, see text and [83] and [84]. (**D**) Autoencoder net. This is a standard MLP without added bias. Its input and output vectors are of the same length and it contains a much smaller ‘bottleneck’ layer. Overall the network serves to output the closest inputs to those on which it has been trained, even when those inputs are then subjected to noise. The values of the outputs of the bottleneck layer form a latent representation of the inputs.

The MLP and RBF architectures of Figure 3 used vectors as their inputs, and while it is possible to unfold a 2D matrix (such as an image) pixel by pixel, row-wise or column-wise, to form a vector, this would lose their natural spatial arrangement. (Note that colour pictures with three colour channels RGB actually form a tensor). Similarly, the number of weights (if one weight was used per pixel) would immediately become infeasibly large. Indeed a tiny 10 × 10 image matrix whose pixels could be just black or white ({0,1}) admits 2^100^ (∼10^30^) variants, so some kind of feature extraction is always required [75]. Another of the pioneers of deep learning, Yann LeCun, long recognised the potential utility of ANNs in image recognition [76], and since their invention has been heavily involved in many of the developments of CNNs. Broadly (Figure 4A), a CNN uses a small matrix (e.g. 5 × 5) to range over the larger image, acting as a filter, and passing its output to the next layer, as in an MLP. Following this, the convolution layers are pooled. This strategy decreases both the number of weights and the likelihood of overtraining. Usually, several layers of convolution and pooling are applied, before a final, fully interconnected output layer. Otherwise, training is fairly conventional, using backprop to adjust the weights. They are widely used in image processing, e.g. in radiology [77], cytometry [78], and breast cancer screening [79]. In general, it is considered that the role of the various convolution and pooling layers is precisely to extract and combine features in abstract form (for image recognition tasks it captures the high-level representation of colour, shape etc. in different layers). Some tricks and tips to train CNNs are at https://stanford.edu/∼shervine/teaching/cs-230/cheatsheet-convolutional-neural-networks.

Thus, AtomNet [80] used vectorised versions of 1 Å 3D grids placed over co-complexes of target proteins and small-molecules bound to them that had been sampled within the target's binding site to effect structure-based drug discovery. This said, a simpler representation of a molecule is as a graph (where atoms and bonds become nodes and edges), and there is an equivalent version of a convolutional network for these called, unsurprisingly, a graph convolutional network or GCN [81,82].

## Recurrent neural nets (RNNs)

Thus far, we have looked only at simple feedforward networks, in which the output of neurons in a given layer acted as input only to neurons in the next layer. While CNNs are arguably the deep network of choice for image processing, many problems such as natural language processing use discrete words in specific sequences. Where the next character (word) is depends on the previous character (word) in a particular word (sentence); in a molecular generation task, the next SMILES character depends in part on the previous character. So instead of a simple input–output transfer function, we need a hidden state with which to try to capture the previous character's information. Here a different kind of architecture is preferred, involving connections between non-adjacent layers, to nodes in the same layer including themselves, and even backwards connections (hence recurrent). A very simple version is shown in Figure 4B. Clearly the number, nature and extent of the ‘recurrent’ connections can be varied considerably. There are different types of RNN to tackle different types of problem with respect to input and output size. These include one-to-many (one input to many outputs) for molecular property to SMILES string generation; many-to-one (multiple input to one output) for SMILES string to prediction of a particular property; and many-to-many (multiple inputs to multiple outputs) e.g. for SMILES character to different molecular properties prediction. The standard backpropagation algorithm has to be modified here, since in unfavourable cases the backpropagation of error could simply be short-circuited. In this case a variant known as ‘backpropagation in time’ is usually used. Even this does not always train well, due to gradient overflow and underflow. However, one specific type of RNN that has come to prominence makes use of a technique that goes back to 1997 [83] but was initially little exploited, known as long short-term memory networks.

## Long short-term memory nets (LSTMs)

LSTMs [83] are a specific type of RNN, and arguably the presently most favoured solution for appropriate problems in deep learning, in which an extra kind of ‘memory’ is explicitly included that effectively helps the recurrent network ‘remember’ its own states from previous epochs during learning, which may include sub-sequences of textual data or previous patterns in time-course data. LSTMs contain special cells (memory cells) with a number of extra parameters that control the functioning of the ‘gates’ (shown in Figure 4C) in the memory cell c_j_ indicated. The multiplicative gate units open and close access to the flow of constant errors. These extra parameters, not all of which are used in every implementation, provide a very considerable richness of behaviour, whose mathematical intricacies we do not develop further here. They have considerable potential in drug discovery [84]. Recent reviews are at [85,86]. The amount of useful ‘memory’ an LSTM can effectively store is rather limited, and improving this is a particularly active area (see e.g. [87]). A similar RNN known as Gated Recurrent Units (GRUs) [88] has fewer parameters than does LSTM; it has been used in [27] for novel molecule generation.

## Autoencoder (AE) nets

The last network architecture of immediate interest to us here (Figure 4D) is the autoencoder. On the face of it this is just a simple MLP with one hidden layer whose input and output vectors have the same length, and where it is intended that the weights are evolved to make the output as close to the input as possible. Crucially, the hidden layer is a ‘bottleneck’ layer; what this does is effectively to force the network to abstract the core signal from the input and this makes it resilient to noise in the inputs. The values of the outputs of the bottleneck layer thus provide a representation of the input of a much lower dimensionality, effectively compressing the data, and these may indeed be used for feature representation, clustering, similarity assessment, or data visualisation (somewhat as in Figure 2). This is effectively a kind of principal components analysis (PCA) when there is only one layer with a linear transfer function. Training is normally via standard backpropagation, as in MLPs.

## Variational autoencoders (VAEs) and generative methods

**Figure 5.**
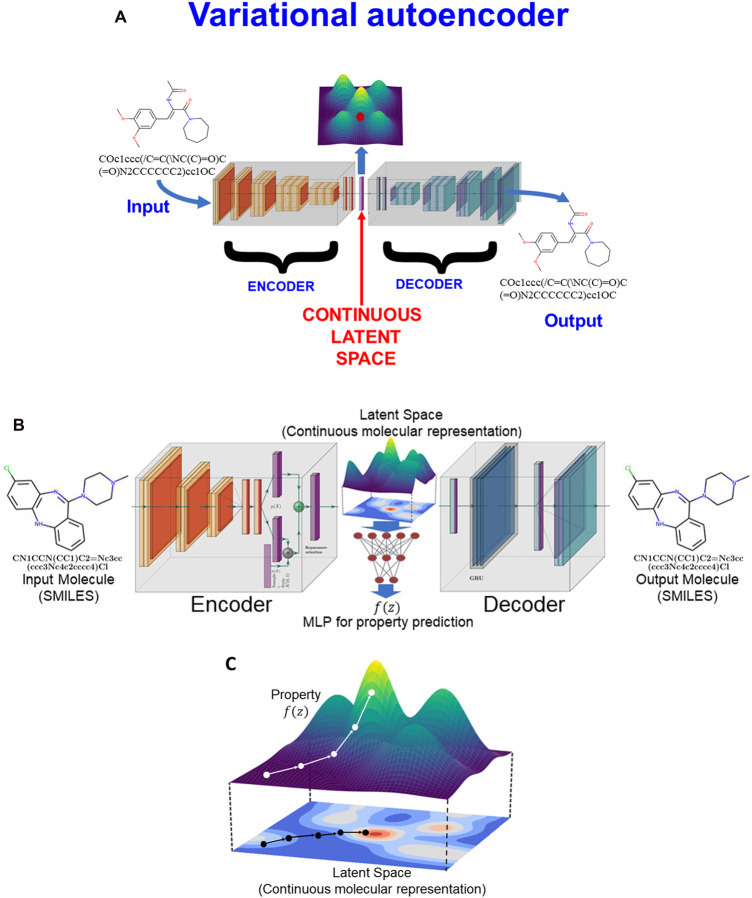
Variational autoencoder networks and their uses. (**A**) Basic VAE architecture, showing the latent space. (**B**) VAE as proposed by Gómez-Bombarelli and colleagues [27]. The latent space is shown as a 2D space for ease of visualisation, but in the paper had a dimensionality of either 156 or (more commonly) 196. (**C**) Moving around the latent space, one simultaneously comes into the ‘basin of attraction’ of particular molecules, whose structures may be output and properties may be calculated via the MLP shown in (**A**) and described in the text (based on [27]). Using optimisation strategies such as evolutionary algorithms can guide the search for the properties and hence the ‘novel’ molecules.

Closely related to autoencoder nets in their appearance (but not at all in their underlying mathematics) are variational autoencoders (Figure 5A) [89–91]. Much of the previous discussion, based implicitly on QSARs, was about what are referred to as discriminative models, where one seeks to learn a predictor from observations (of paired molecules and their activities). What we would really like to have is the ability to generate the molecules themselves ‘*de novo*’ (e.g. [59,64,65,84,92–107]), by learning what amounts to a joint distribution over all the variables (both inputs and outputs). To this end, a generative model seeks to simulate or recreate how the data are generated ‘in the real world’. Generative models can be trained on existing data and used to generate novel text, images and even music. For our purposes, a generative model can learn to generate molecules that it has never ‘seen’ during the training phase. Of course this means in principle that we can go from the comparatively modest number of molecules that have ever been made and contemplate sampling (generating) them from the much more massive universe (described above) of molecules that might be made. A VAE (Figure 5) consists of two main halves: an encoder (sometimes called a recognition model), and the decoder (which is the generative model). Joining them is a vector (which may be, but does not have to be, of lower dimensionality) that represents the latent space between the two (Figure 5A). A VAE effectively learns stochastic mappings between an observed (input and output) space, whose distribution is provided by the real world, and a latent space that is purposely much simpler and is continuous. Deep learning VAEs are those that are trained using general neural network principles and have multiple ‘hidden’ layers for both the encoding and the decoding. The particular recognition here is that we need to move between the discrete space of molecules (often encoded as SMILES strings [108], but increasingly as molecular graphs) and the continuous space of the neural networks and latent variables of the autoencoders. Fortunately this is now possible, using techniques such as molecular grammars [109,110], and direct graph generation [111–113]. We note that SMILES strings present particular difficulties because their grammar is context-sensitive: making a cut in an arbitrary place in a SMILES string (in contrast with doing so in a protein sequence represented in the one-letter FASTA amino acid code) does not normally lead to two fragments with valid SMILES. (One potential solution to this is to adapt the SMILES grammar to remove the most troublesome elements; DeepSMILES [114] is an example of this.).

### A specific example

A particularly clear example of the utility of generative methods is provided by Gómez-Bombarelli and colleagues [27] (Figure 5B), who encoded and decoded SMILES strings and represented them using a VAE. Since they could easily calculate molecular properties from the SMILES (using RDKit, www.rdkit.org), they also trained a standard MLP to use values of the latent vector as inputs and the calculated properties as outputs. In principle, any kind of deep network might be used for the encoding, and the same or any other kind for the decoding [115]. In this case, the input (encoder) network [27] was mainly a CNN while the output used a specific type of RNN called a gated recurrent unit [116,117]. The latent space used [27] was mainly of 196 dimensions, and the VAE was trained to reproduce its inputs at the outputs (another module from RDKit was used to filter invalid SMILES strings). (Parenthetically, the inputs to be encoded could have been InChI and the outputs decoded as SMILES [118]!)

Now the magic happens. Armed with the ability to generate SMILES strings (and hence molecular structures) from the latent space, the authors could then either perturb the values of latent space vectors away from known molecules, or pick more-or-less arbitrary vectors, see what SMILES strings were generated by the trained decoder, and simultaneously estimate the molecular properties of interest (Figure 5B,C). This allowed them to generate valid molecules with desirable properties (although they did still generate many non-valid SMILES strings). Having a continuous latent space linked to molecular properties (as well as molecular structures) turns the search into an optimisation problem (Figure 5C); many methods are available for such problems [9], and they chose an evolutionary algorithm.

The power of these methods meant that they could also generate chemically valid but potentially bizarre molecules, so the objective function was varied to include high values for the quantitative evaluation of drug likeness [119] (QED) and synthetic accessibility [120] (SA) scores. Although they trained their VAE using only some 250 000 molecules from the ZINC database, they estimated that they could find (i.e. generate) 30 different molecules from any training point in the latent space, implying that their VAE had effectively ‘stored’ the ability to make predictions about 7.5 M molecules. Although there is no simple mapping, molecules that are encoded by vectors that are ‘close’ in the latent space may be expected, to some degree, to be closer in structural space as judged by conventional cheminformatic fingerprints and Tanimoto distances, and hence will tend to have similar properties [121]. Understanding the extent to which this is true this will be of considerable value in the future. Plausibly, even larger nets (or ones with different architectures) trained on much bigger datasets would have generalised to many more molecules. Indeed, Arús-Pous and colleagues [122] trained an RNN with 0.1% of the 675-million GDB-13 database and established that it could generalise to create nearly 70% of GDB-13 while sampling over 2 Bn molecules.

**Figure 6.**
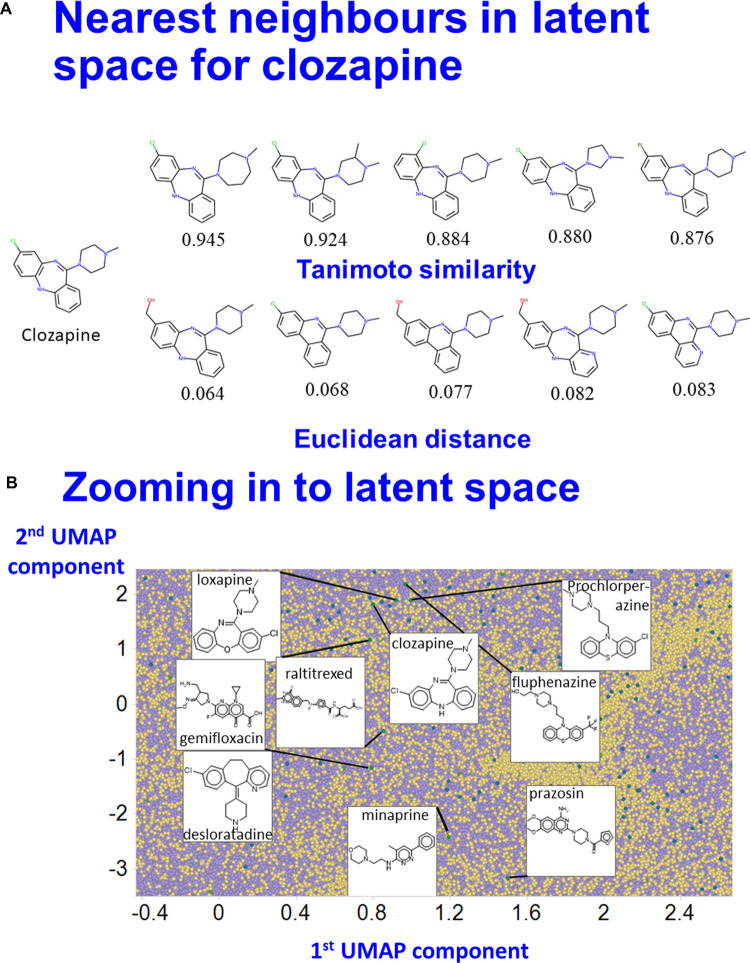
Local properties of latent space. (**A**) Molecules ‘near’ clozapine. A VAE was trained as in Figure 2 and then the vector representing clozapine modified slightly. The closest five molecules generated are shown, as judged either by the Euclidean distance of the random modification or the Tanimoto similarity (RDKit patterned encoding). (**B**) Moving between two selected drugs (here clozapine and prazosin), it is clear that they share many obvious (and possibly non-obvious) structural similarities. UMAP components and labelling are as in Figure 2 save that selected drugs are here shown with green symbols.

Finally, Figure 6 shows two of these properties of generational networks, using the same VAE as used in Figure 2. In Figure 6A, we take the molecule clozapine that has a particular position in the 100-dimensional latent space. We then perturb this vector randomly by a small amount, read the SMILES strings generated by the decoder (filtering out those that are invalid), and return the molecules so generated. Note that none of these molecules was presented to the network at any point (training, validation or test). Of course ‘similarity’ is in the eye of the beholder, and of the encoding [123], but the top 5 molecules so generated are also ranked and labelled with the Tanimoto similarity to the original molecule as assessed using the RDKit ‘patterned’ encoding, and appear sensible. Similarly, Figure 6B shows some of the drugs ‘encountered’ on a trip between clozapine and prazosin somewhere near the centre of the space depicted in Figure 2. Again, if one is interested in either of these one might then assess such drugs in any assays of interest, having narrowed the search space considerably.

## Methods to improve generalisation

As mentioned, really the key concept in neural networks is their effective ‘storage’ of knowledge as matrices of weights, and their ability to generalise to unseen inputs that are at least reasonably related to those used in their training. Although described in various ways, the bane of neural network training hinges on their tendency to overtrain, i.e. to overfit the training data while losing the ability to predict accurately when presented with new data. Virtually all remedies are designed to minimise this and thus to improve generalisation by ‘sparsifying’ or regularising the trained network. Some such methods include the use of ‘decoys’ (especially generative adversarial networks [53,124,125]), the use of heavy pruning during training, especially ‘dropout’ [126], training in small batches [127], using ensembles of the same network [128], and the addition of noise (even ‘randomised’ SMILES strings [71,117]) to the inputs. It seems that in some cases the values of the hyperparameters are critical, and they interact with each other in complex and hard-to-predict ways (and they may also be optimised using evolutionary algorithms). This makes their training computationally demanding, even before starting to vary the architecture (known as neural architecture search [129–133]). Clearly, there is no limit to the possibilities with which one might combine network architecture modules. Increasing model capacity can either improve or hurt generalisation, depending on the difficulty of the generalisation problem, though the density in latent space is a key determinant of the difficult of a generalisation task [134]. There is a trend towards very large networks that (perhaps unexpectedly [135]) do not overtrain [55]. The biggest and most successful deep networks, presently GPT-3 [55], use transformer [136] architectures, including in drug discovery [137,138]. The largest flavour of GPT-3 has 96 layers with 12 299 nodes in each. At this stage we are not aware of even empirical rules relating e.g. the size of the latent space and the size of the training set of molecules, so an exploratory process, tuned to the problem of interest, is suitable.

## Some further examples of deep and/or generative networks in chemical biology

The ability to ‘generate’ molecules *in silico* is in many ways the most exciting of the possibilities opened up by these new methods, since this allows an attack on the ‘inverse’ problem [139] highlighted at the beginning, i.e. to find novel molecules with desirable bioactivities even if those molecules do not themselves appear in existing databases (and of course if they do). Such molecules may not even previously have been synthesised. This is a very active area, so we give just a few examples.

### Antibiotic discovery

Antimicrobial resistance is a well-known and major problem, and it would be desirable to find new anti-infectives (e.g. [140–142]). Thus, Collins and colleagues [143] trained a deep neural network on 2335 molecules that had been shown experimentally to inhibit the growth of *E. coli*. The trained network was applied *in silico* to a variety of other libraries, suggesting that a number of apparently unrelated (and untested) molecules, ones quite different in structure from known antibiotics, might prove to be of value as anti-infectives. One, named halicin (5-{(5-nitro-1,3-thiazol-2-yl)sulfanyl}-1,3,4-thiadiazol-2-amine), was shown to have a broad spectrum of antibacterial activity and appears highly promising. Although halicin is far from their structures, it is noteworthy that nitrofurantoin and metronidazole are two other existing antibiotics with a nitro group on a five-membered heterocycle, and whose mode of action means that resistance is both rare and hard to come by. Specifically, these kinds of molecule seem to have multiple transporters and are reduced to active, radical species.

One feature of *in silico* analyses is their potential for speed. Nowhere is this more obviously desirable than in the search for safe and effective molecules against coronaviruses, especially that (SARS-Cov-2) causing COVID-19. Shin et al. developed a deep learning strategy termed MT-DTI [144], which they trained on (the then) 97 million molecules in PubChem, and found that they could accurately predict binding affinities based on small molecule structures (transformed from SMILES) and the FASTA amino acid sequences of target proteins within bindingDB. Based on this, Beck and colleagues [145] could predict the potential activity against SARS-CoV-2 of a variety of known antivirals.

### Drug discovery more generally

The availability of very large experimental datasets [62,146], both online and within companies, will clearly enable much better kinds of virtual screening to be performed, as properties do not simply have to be calculated but can be measured. This said, the increasing power of computers is also bringing forward the ability to calculate many more properties of interest via (time-dependent) density functional theory [147].

Gupta et al. [148] trained a variety of LSTMs in generative molecular discovery, with great success, training on 550 000 SMILES strings from ChEMBL, and generating over 25 000 novel molecules, and many others by growing fragments. Ertl et al. [149] and Segler *et al*. [150] have used similar strategies for similar purposes, and showed that such methods can perform the complete *de novo* drug design cycle to generate large sets of novel molecules for drug discovery. Yasonik [97] combined *de novo* molecular generation *in silico* (using RNNs) with a multi-objective evolutionary algorithm in an iterative method for selecting suitable molecules subjective to constraints on their physicochemical properties. Finally, here, our own laboratory has developed methods [151] based on molecular graphs and reinforcement learning for generating molecules predicted (from existing binding assays) to have a specific set of differential activities; the methods are entirely general.

## Other areas

For reasons of space, we do not cover in detail a variety of other deep learning areas that may be of interest to readers. However, we give some outlines of strategies in chemical syntheses [152–154] and protein structure prediction [155,156], as well as in optimisation, where deep learning methods are also enjoying considerable success.

### Chemical syntheses

Using ‘intelligent’ methods to decide which molecule is of interest is one thing (probably some 10^6^–10^7^ are easily commercially available, depending on the latency of that availability). Using intelligent methods to choose a means by which to make them oneself is entirely another [152–154,157–162]. Probably the present apotheosis of this strategy is Chematica [157,158,163] (recently commercialised as Synthia^TM^), that started life as a manually encoded set of reaction rules and now uses (so far as one can tell from published material) a variety of means of intelligent search. The exciting prospect is for the discovery of entirely novel reactions based on those presently known; this is precisely the area in which generative methods can excel.

### Protein structure prediction

Leaving aside those that need so-called chaperones, proteins are formed of strings of amino acids (primary structure) that fold up spontaneously to produce the active form of the protein (tertiary structure), which is itself assembled from the coming together of recognisable motifs that include secondary structures such as α-helices and β-sheets. Uniprot https://www.uniprot.org/ (that houses the downloadable set) presently contains some 180 M nonredundant natural protein sequences, increasing at roughly 25 M p.a. (https://bit.ly/2MZekYJ). With an average length of 337 residues, these represent ∼60Bn amino acids. In contrast, the protein databank https://www.rcsb.org/ contains only some 170k 3D structures; since sequencing speeds are far greater than are the methods of structure determination the gap is inevitably going to grow. Consequently, there has long been considerable interest in predicting structure from sequence. Correspondingly, the existence of so many sequences allows the use of unsupervised methods to help to populate the deep learning systems that can then be exploited using supervised methods for the fewer structures there are. Thus, Google DeepMind and collaborators developed Alphafold to optimise the interatomic potentials that control this assembly. They did so by combining three methods: (i) Memory-augmented simulated annealing with neural fragment generation; (ii) memory-augmented simulated annealing with neural fragment generation with distance potential, and (iii) repeated gradient descent of distance potential [156,164]. If the improvements seen during the evolution of their Go-playing reinforcement-learning-based programs [54,165,166] are anything of a guide, we may soon anticipate considerable further improvements. Similar comments might be made about the activities of specific protein sequences [167–170].

### Optimisation

Most scientific problems can be cast as combinatorial search problems (‘find me the best experiments to do next out of a potentially vast number') [9]. Even in a simple ‘static’ case where each element can take just M values in an array N of possible parameters, this scales as M^N^ (e.g 4 bases in a string of 30 = 4^30^ ∼ 10^18^ possibilities [171]). This clearly applies to problems in chemistry and chemical biology that involve navigating large search spaces of molecules, and intelligent automation has been an important subset of activities here (e.g. [172–182]). ‘Active learning’ describes the kinds of methods that use knowledge of existing data to determine where best to explore next, and is normally used to balance exploration (looking for promising regions of the search space) with exploitation (a promising local search) [183]. Of the many strategies for this, evolutionary (‘genetic’) algorithms are pre-eminent, and the area is sometimes referred to as ‘inverse design’ [64]. Where the deep learning meets them is illustrated by the work of Nigam et al. [184], who used a genetic algorithm plus a generative model to optimise the ‘penalised’ logP (J_m_), where J_m_ = logP + SA + RingPenalty and in which logP is the octanol:water partition coefficient, SA is a synthetic accessibility score [120], and RingPenalty adds a penalty for rings larger than six atoms. Other areas have involved organic light-emitting diodes [147] and redox-based batteries [185]. Clearly these methods are generic, and can admit any objective function that can be calculated or measured.

Another important area of optimisation is in microbial biotechnology, whether in finding the best growth medium [186], subsets of genes to manipulate to increase productivity [187,188], or optimal sequences for generating host [189] or protein properties [190,191]. Each of these represents a combinatorial search problem [8,9].

Our last example here involves the search for an optimal (signalling) sequence for effecting protein secretion. Although secretory signals of 15–30 amino acids are more or less well known for systems such as *sec* [192] and *tat* [193] in *E. coli*, natural evolution seems to have performed only a rather limited and stochastic search of these quite large sequence spaces. Thus, Arnold and colleagues [194] used deep learning methods to model known sequences, and could predict novel ones that were ‘highly diverse in sequence, sharing as little as 58% sequence identity with the closest known native signal peptide and 73% ± 9% on average' [194]. These kinds of findings imply strongly that because Nature tends to use weak mutation and strong selection [8], necessarily becoming trapped in local optima, much is to be gained by a deeper exploration of novel sequence spaces.

## Interpretability

A widespread and reasonable criticism of these ‘deep learning’ methods is that while they may be good at predicting interesting things, the means by which they do so is entirely opaque [195]. Unravelling this is known as ‘interpretable’ or ‘explainable’ AI. A common view is that ‘disentangling’ the inputs in the encoder of a VAE will lead to a representation in which individual features (or small subsets of features) of the latent space more nearly approximate features of the inputs (e.g. [196–200]). Many flavours do this by adding regulariser terms to the output objective function [201–203], such as in β-VAE [204,205], Deep VIB [206], PixelVAE [207], InfoVAE [208], PRI-VAE [203], VAE-LIME [209], Langevin-VAE [210], Discond-VAE [211], and Gaussian mixture VAE [212], while supervised methods also help with interpretability [213]. The ability to improve our understanding of which parts of a molecule are important for its activity (the ‘pharmacophore’) is particularly prized in drug discovery [214].

## Looking to the future

Although the future of these kinds of methods is very encouraging generally, it is clear that there are a number of fruitful directions to be explored, beyond the obvious one of novel and better deep learning architectures, algorithms and hyperparameters. One is the representation of small molecules [215], a key to all computational methods, in both 2D and 3D; here the SELFIES approach [216] appears very promising. Another is the use of entirely hardware architectures; these are called neuromorphic computers, and include the spiking neural network engine SpiNNaker [217]. Thus, neural networks effectively amount to (and might loosely be modelled as) electrical circuits. One electrical component that was only recently rediscovered is the memristor; they have been badly underexplored as elements in ANNs but it is easy to predict that this is likely to be an area of fruitful activity [218].

Another area that we find surprisingly underpopulated is that of neuroevolution. While backpropagation is both popular and reasonably well understood, and may even be used in biological learning [219], it is still rather slow. However, adjusting weights (and even architectures) in deep networks to reach a desirable outcome is simply another kind of optimisation problem, that is perfectly amenable to the many flavours of evolutionary computing that work very effectively in optimisation. This has long been recognised [220,221], and since evolutionary algorithms are mostly ‘embarrassingly parallelisable’, we may anticipate considerable advances in the use of neuroevolution for deep learning in chemical biology.

Much of the current activity uses labelled datasets, but far more unlabelled datasets are available. It has been predicted (not least since human learning is mainly unsupervised) [52] that unsupervised learning will play a much larger role in the future, and this seems reasonable. Self-supervised methods [32] seem to show particular promise.

It was always a reasonable view that the much greater ability of humans than machines to store sense data and to reason about them was largely predicated on the simple fact that human brains contain many more neurons. This does now seem to be the case, as very large nets, showing a clear appearance of natural language ‘understanding’ (e.g. [55,222]), become available. They do, however, require very considerable computational resources to train, even with modern GPU-based (and similar) hardware.

With regard to chemical biology, the original motivation of the ‘robot scientist’ project [172] was in fact to conceive and synthesise small molecules; the generative abilities of deep networks now seem capable of bringing this fully closed loop activity within reach [152]. Furthermore, understanding the relationships between molecules that are encoded by vectors in the latent spaces used by deep networks, and their closeness to each other in structural and bioactivity spaces, is likely to be of much value.

From a scientific point of view, the empirical success of deep learning comes at a major cost. That cost, as mentioned, is the cost of knowing precisely how the ‘knowledge’ is actually stored in a deep network, and thus exactly how inputs map to outputs . It is also assumed that such knowledge will help avoid costly and dangerous errors as we begin to trust these methods more. Thus, ‘explainable AI’ will continue to be an important area for the future.

## Concluding remarks

This has been a purposely high-level overview of some of the possibilities in cheminformatics and chemical biology that have been engendered by the development of deep learning methods in general and of generative methods in particular. Our main aim has been to draw attention to these developments, and to some of the means by which readers who are only loosely acquainted with them can incorporate these methods into their own work. Its success will be judged by the rapidity with which this happens.

**Glossary**

- **Discriminative model.** A type of ‘supervised’ machine learning model used for classification or regression problems, based on the learning of relationships between pairs of inputs X and outputs Y. Such models serve to create decision boundaries in high-dimensional space, that allow the prediction of new outputs given new inputs of comparable type. They allow modelling of the probability of Y given a value of X (P (Y|X = x)). In chemical biology they are familiar as QSAR models. Most classical ML methods involve discriminative modelling.
- **Generative model**. A much more powerful (and difficult) approach to modelling that also relates inputs X to outputs Y but that allows the *de novo* prediction of X given Y. They can do so because they capture the joint probability distribution p(X, Y) over the training data, and effectively permit the prediction of candidate inputs X given a desired output Y (i.e. P(X|Y = y)). Generative models have also been used to great effect in a very particular way in deep learning to create ‘fake’ or ‘decoy’ inputs, whose avoidance can assist deep networks in learning to generalise better [53,124]. These are commonly referred to as **Generative Adversarial Networks** (GANs).
- **Generative Adversarial Networks (GANs)**. It was recognised by Ian Goodfellow [53,124] that one way to help generative networks generalise better was to try to fool them with ‘fake’ examples (initially random data representing images). This has been both massively successful and highly popular, with many new flavours becoming available.
- **Hyperparameters**. The training of neural networks can be affected greatly by many control variables including the size, number and connectedness of layers, means of weight initialisation, learning rate, momentum, weight decay, transfer function, and so on. These hyperparameters still tend (and need) to be adjusted empirically for specific problems. Tuning hyperparameters is itself an optimisation problem, best addressed via intelligent search.
- **Overtraining**. Equivalent to overfitting a function by adding too many terms, this is equivalent to learning a model that fits the training data perfectly but is less good at fitting the validation or test data. It is the greatest danger of ANNs of all stripes. Indeed, almost every strategy for improving training is ultimately designed to decrease the overfitting of the training set.
- **Radial basis function networks**. These networks have a broadly similar architecture to MLPs (Figure 3B) save that the initial training consists of the application of an unsupervised (clustering) method that assigns the midpoint and width of a series of (commonly Gaussian) radial basis functions based on the examples, then uses a linear fitting between the RBFs and the output layer to train the input–output mapping.
- **Supervised learning**. One of the four main types of machine learning (the others are unsupervised, semi-supervised, and reinforcement learning). This uses paired inputs X and outputs Y to learn a nonlinear mapping from one to the other. In **unsupervised learning** the class membership of the outputs Y is not given, so only clustering is possible, while in semi-supervised learning some (usually a small subset) of the class memberships (outputs) are known and may be used to guide the learning. In reinforcement learning, a software agent takes actions in an environment, which is interpreted into both a reward and a representation of the state; these are fed back to the agent and may be used iteratively in a decision-making process. Reinforcement learning underpins many of the great successes of Google DeepMind, such as in Go playing [54,166].
- **Universal approximation**. A very powerful theorem that shows that any feedforward network with just a single hidden layer (of unstated and hence arbitrary size) and a suitable, nonlinear transfer function can effectively approximate anything from a continuous distribution. Although perhaps a little overblown, it does provide the conceptual underpinnings for much of the power of ANNs.

## Open Access

Open access for this article was enabled by the participation of University of Liverpool in an all-inclusive Read & Publish pilot with Portland Press and the Biochemical Society under a transformative agreement with JISC.

## Abbreviations

ANN: artificial neural network
CNN: convolutional neural network
GRU: Gated Recurrent Units
LSTM: long short-term memory
MLP: multilayer perceptron
QSAR: Quantitative structure-activity relationship
RBF: radial basis function
ReLU: rectified linear unit
RNN: recurrent neural network
VAE: variational autoencoder
